# Sensory processing sensitivity and somatosensory brain activation when feeling touch

**DOI:** 10.1038/s41598-022-15497-9

**Published:** 2022-07-14

**Authors:** Michael Schaefer, Anja Kühnel, Matti Gärtner

**Affiliations:** Medical School Berlin, Rüdesheimer Str. 50, 14197 Berlin, Germany

**Keywords:** Psychology, Human behaviour, Personality, Neuroscience, Cognitive neuroscience, Sensorimotor processing, Sensory processing, Social behaviour, Social neuroscience, Somatosensory system

## Abstract

Sensory processing sensitivity is described as a personality trait associated with a high sensitivity to environmental and social stimuli. It has been assumed that about 15–20% of the total population can be described as highly sensitive. The concept states that those individuals represent a higher sensitivity to subtle stimuli, thereby exhibiting a different somatic sensation. Here we aim to test the assumption that the brain’s sensory perception is different in individuals with high sensory processing sensitivity. We used a German version of the Highly Sensitive Person scale to measure sensory processing sensitivity. Furthermore, we assessed the Big Five personality dimensions and trait empathy (using IRI). To test the hypothesis that the brain’s handling of sensory information is different in individuals with high sensory-processing sensitivity, we scanned participant’s brain activity with functional magnetic resonance imaging (fMRI) while they were touched by an experimenter’s hand. Results showed positive correlations of sensory processing sensitivity with neuroticism, openness, and empathy. Introversion was not a significant predictor. Neuroimaging data demonstrated that sensory processing sensitivity (controlled for associated personality dimensions) was not related to primary or secondary somatosensory BOLD responses, but positively associated with BOLD activity in left posterior insular cortex. Based on these results we conclude that sensory processing sensitivity seems to represent insula-mediated affective touch. We discuss these results with previous studies reporting an engagement of the insula in individuals with high sensory processing sensitivity.

## Introduction

Each day we are faced with numerous stimuli, which our brain has to process and integrate into a coherent representation of the world. It seems likely that there may be individual differences in the way our brains handle this challenge. These differences have been described in theories on environmental sensitivity^1^. One example of these approaches is the concept of sensory processing sensitivity (SPS)^2^. This theory argues that individuals differ with respect to their sensitivity both to aversive and supportive environmental stimuli (leading some researchers to call extremes of these personalities as orchids and dandelions, e.g.^3^). It proposes (in contrast to other theories on environmental sensitivity) that these differences have to be understood as an expression of a personality trait (not a disorder)^4,5^.

How is the personality trait SPS described? According to Aron et al. individuals with high SPS have greater sensitivity to environmental and social stimuli. They show higher emotional reactivity and are described to have greater depth of processing, which has been supported by research on visual processing sensitivity^6^ . The characteristics of SPS also include higher empathy. Moreover, they are described to be more sensitive to beauty and art^2,5,7^.

The first and most established questionnaire to measure SPS is the Highly Sensitive Person scale (HSPS)^2^. It consists of 27-items and has been validated by several studies (e.g.,^8,9^). Although originally developed as an unidimensional questionnaire, recent psychometric studies fitted models with three factors: ease of excitation, aesthetic sensitivity, low sensory threshold^9,10^. The SPS includes items, for example, as “Are you easily overwhelmed by strong sensory input?” or “When you were a child, did parents or teachers seem to see you as sensitive or shy?”.

When comparing the SPS to the established Big Five model of personality a strong relationship to neuroticism and openness has been consistently reported. In addition, introversion has been linked to SPS^3,11^. However, according to Smolewska et al., the concept of SPS cannot be fully explained by those traits^10^. For example, it has been shown that neuroticism has only a medium level magnitude of correlation with SPS, suggesting that SPS is distinct from neuroticism^10,12^. Furthermore, research suggested that differences in SPS may be genetically based^13^.

In addition, it has been hypothesized that SPS is associated with empathy^2^. However, there are only few studies that directly address the link between empathy and SPS^14^. In particular, there seems to be a lack of studies examining correlations with SPS and the personality trait empathy.

Although the theoretical concept of SPS is biologically founded, only few imaging studies investigated the neural basis of this personality trait. Acevedo et al. employed an fMRI approach to examine reactivity of participants towards photos of their romantic partners and of strangers showing positive, negative, or neutral face expressions. They report stronger activations in brain regions engaged in awareness, empathy, and self-other processing (cingulate, insula, inferior frontal gyrus, middle temporal gyrus) for individuals with high SPS when watching pictures of their partners or of happy faces^15^. Wu et al. examined resting state activity to analyze the relationship between SPS and depression and found that a subdimension of the SPS, ease of excitation, correlated with grey matter volumes in cerebellum and right dorsal anterior cingulate cortex (ACC)^16^. Jagiellowicz et al. focused on neural responses to subtle changes in visual scenes^6^. They showed that SPS was associated with stronger activation of brain areas engaged in higher-level visual processing. Aron et al. investigated the interaction of SPS and cultural background. They reported differences in brain areas involved in attention and working memory that are moderated by SPS^7^. Acevedo et al. examined resting state activity after participants completed a social affective empathy task. They found increased brain connectivity in regions representing attentional control and consolidation of memory for individuals with high SPS. Based on these results the authors conclude that depth of processing seems to be central for SPS^14^.

Almost all of the imaging studies on SPS focused on visual stimuli, whereas other modalities such as the tactile modality have been neglected. This is surprising, given that the tactile modality is one of the first senses we develop and even invertebrates seem to own this sense to contact the world. Moreover, the tactile domain is particularly important when considering social stimuli, which seems to be important for SPS. The aim of our study was to address this gap by examining the neural underpinnings of SPS when processing touch. While we scanned their brain activity, participants simply received touch by the hand of an experimenter. Based on the SPS theory we hypothesized that the strength of activity in somatosensory cortices would be associated with the magnitude of SPS. More in detail, we assumed correlations with SPS in primary somatosensory cortex and insula, which have been shown to be involved in social perception (e.g.,^17–19^). Moreover, the insula seems to play an important role for affective touch, as demonstrated, for example, by Olausson et al.^20^.

Before starting the imaging experiment, we conducted a behavioral study to examine how much Big Five personality dimensions and empathy contribute to the concept of SPS (N = 165). In a second step, we then examined brain responses of participants (N = 22) to test the hypothesis that high SPS is reflected by stronger brain responses in somatosensory brain areas when feeling touch on the palm of the hand. The first step seems necessary, since we need to control personality traits that are linked with SPS when investigating possible correlations of touch-related brain responses.

## Materials and methods

### Participants

The first part of our study examined 165 German participants (113 females, mean age 29.17 ± 11.32 years). The second part of the study used fMRI and included 22 German participants (17 females, mean age 21.38 ± 2.89; thirteen subjects were randomly taken from the first study). None of the participants had a neurological or psychiatric history. The study was approved by the ethical committee of the Medical School Berlin (Germany) and adhered to the Declaration of Helsinki. All participants gave written informed consent to the study.

The datasets analyzed during the current study are available from the corresponding author on reasonable request.

### Procedure

All participants were asked to complete questionnaires on SPS (HSPS-G^2,9^), personality (NEO-FFI, Costa^21^), and empathy (IRI, Davis^22^).

To measure SPS we used a German version of the HSPS that has been developed by Aron et al.^2,23^ (see supplementary data). The HSPS scale includes 27 items and is widely used to measure SPS.

Personality was measured based on the five-factor model. We used a German version of the NEO-FFI, an established questionnaire to measure the Big Five personality dimensions^21,24^. It includes 60 items to describe the human personality in five core dimensions: neuroticism, extraversion, openness, conscientiousness, and agreeableness. The dimension neuroticism is linked to negative emotions such as anxiety and irritability. Extraversion is related to sociability, assertiveness, and talkativeness. Openness to experiences is displayed by aesthetic sensitivity and intellectual curiosity. Conscientiousness describes disciplined and organized behavior. Agreeableness is described as a tendency to altruism and politeness^21^.

Empathy was measured with the SPF, which is a German version of the IRI^22,25^. The IRI is widely used and extensively validated (e.g.,^26,27^). It measures self-reported empathic behavior and includes 28 items with four subscales: perspective taking, fantasy, empathic concern, and personal distress. Perspective taking reflects the propensity to cognitively imagine a situation from the other person's point of view. Fantasy measures the participant’s ability to transpose oneself into the feelings and actions of fictional characters in books, movies, or plays. The subscale empathic concern refers to feelings of compassion, sympathy, and concern for others. Personal distress describes the tendency to feel distress or unease when witnessing distress in others^22^.

For the second part of our study 22 individuals participated in an fMRI experiment. While we scanned their brain activity, participants received passive touch by the hand of an experimenter, who was close to the scanner. The experimenter touched the palm of the participant’s right hand (skin area size about 4 to 6 cm) ten times in a caressing way (touch condition), with a frequency of about one touch per second. The experimenter used his fingers (digits 2 to 5) to apply the touch. The control condition was a time window (12 s) where we applied no touch at all.

Subsequently we asked the participants to rate the strength of the felt touch (for two seconds) and how pleasant it felt to them (2 s). Participants responded using a key with four buttons (Likert-scale, 1 = not at all strong/pleasant, 4 = very strong/pleasant). These questions were included in order to test whether pleasantness or perceived strength of the touch was linked to SPS.

There was a break of 12 s after participants responded (= no touch condition). In total, we applied 20 touch (and twenty no touch) blocks in four runs.

### FMRI data acquisition, image preprocessing, and analysis

FMRI data were acquired with 3 T Siemens Tim Trio scanner (Siemens, Germany). BOLD responses were obtained using axial oriented echoplanar T2-weighted images (TR = 2 s, TE = 35 ms, flip angle = 80 degrees, FOV = 224 mm, number of slices = 32, voxel size = 3.125 × 3.125 mm, slice thickness = 3.5 mm). Prior to the functional runs high-resolution T1-weighted structural images were recorded for anatomical reference (MP-RAGE sequence, TR = 1650 ms, TE = 5 ms). Four participants were scanned with an updated system to a Magnetom 3 T Prisma Fit (analogue procedures). Participants were allowed to take short breaks between the runs. We placed foam cushions around the side of the subject’s head to minimize head motion.

Statistical Parametric Mapping Software (SPM12, Wellcome Department of Imaging Neuroscience, University College London, London, UK) was used for data preprocessing and subsequent statistical analyses. Preprocessing steps included realignment to correct for inter-scan movement (spatial realignment to the mean image), coregistration, normalization into a standard anatomical space (MNI, Montreal Neurological Institute template), and smoothing with a Gaussian kernel of 8 mm.

We then calculated statistical parametric maps by using multiple regressions with the hemodynamic response function modeled in SPM. First, we analyzed data on an individual subject level (fixed-effects-model, comparing touch relative to no touch blocks). The resulting parameter estimates for each regressor at each voxel went into a second-level analysis (random effects model). We report active regions at p < 0.05 corrected for multiple comparisons over the whole brain and for anatomically defined regions of interest (ROIs) (family-wise (FWE) corrected). These ROIs were based on the SPM anatomy toolbox and included primary somatosensory cortex (SI), bilateral somatosensory cortex (SII), and bilateral anterior and posterior insula. In addition, we included ROIs of the right dorsolateral prefrontal cortex (DLPFC), ventral ACC, and medial prefrontal cortex (PFC) based on previous studies addressing the relationship with the touching individual^28^ or empathy in SPS^14^.

To test which personality measures explain SPS we analyzed the behavioral data by standard multiple linear regression analyses (all Big Five personality measures and empathy went simultaneously into the model). In addition, the model included sex and age as predictors, because previous studies discussed the influence of these variables on SPS^2,29^.

Furthermore, we calculated peak activations in SI, bilateral SII, and bilateral anterior and posterior insula (as well as other ROIs) and examined the relationship of those brain activations with SPS by using Pearson correlations (controlled for personality measures associated with SPS).

## Results

### Behavioral results

**Figure 1:**
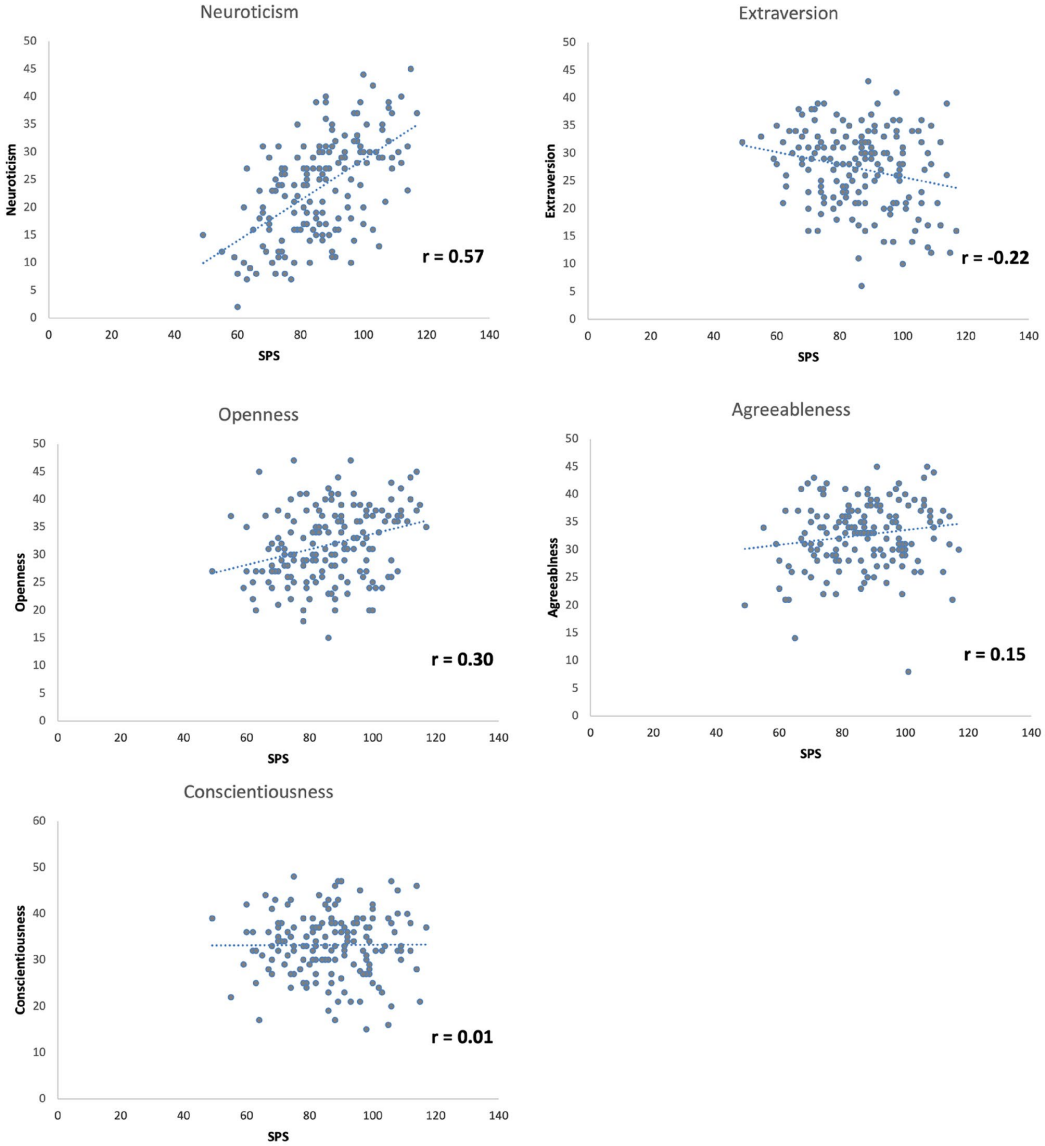
Scatterplots of SPS and Big Five personality measures (NEO-FFI). Results demonstrate significant positive correlations of neuroticism and openness with SPS. Extraversion was negatively related to SPS (Pearson correlations).

Mean scores for SPS, NEO-FFI, and IRI are shown in Table 1. Figure 1 displays the scatterplots of SPS and Big Five personality dimensions. Pearson correlations demonstrate strong correlations of neuroticism and openness and a smaller negative relationship of extraversion with SPS (neuroticism: r = 0.57, p < 0.001; extraversion: r = −0.22, p = 0.004; openness: r = 0.30, p < 0.001; agreeableness: r = 0.15, p = 0.048; conscientiousness: r = 0.01, p > 0.10; two-sided).

**Table 1:** Results of personality questionnaires HSPS, IRI, and NEOFFI.

	Mean ± standard deviation
**SPS**	86.39 ± 14.02
**NEO-FFI**	
Neuroticism	23.64 ± 9.01
Extraversion	27.22 ± 7.08
Openness	31.90 ± 6.57
Agreeableness	32.65 ± 5.99
Conscientiousness	33.26 ± 6.87
**Empathy personality questionnaire IRI**	
Empathic concern	15.05 ± 2.53
Personal distress	11.07 ± 3.03
Perspective taking	15.07 ± 2.61
Fantasy	13.99 ± 3.38

We then tested whether Big Five personality measures (NEO-FFI) and empathy (IRI) explain SPS by means of a linear regression analysis. All five personality measures as well as the global empathy score of the IRI went simultaneously into the model. Furthermore, we included sex and age as predictors. The results revealed a significant model (R = 0.74, adj.R2 = 0.52, F(8,164) = 22.91, p < 0.001). Neuroticism was a strong positive predictor for SPS (beta = 0.55, p < 0.001), as well as openness (beta = 0.21, p = 0.002). A further strong positive predictor for SPS was empathy (beta = 0.25, p < 0.001). Sex and age were no significant predictors (see Table 2).

**Table 2:** Regression analyses of SPS with personality measures as predictors.

Model					Coefficients (standardized)		
R	R^2^	Adj. R^2^	ANOVA		Betas	T	Sign
0.74	0.54	0.52	F (8,164) = 22.91, p < 0.001	Neuroticism	0.55	8.41	**p < 0.001**
				Extraversion	−0.04	−0.63	p = 0.527
				Openness	0.21	3.20	**p = 0.002**
				Agreeableness	0.08	1.31	p = 0.192
				Conscientiousness	0.12	2.01	p = 0.046
				Empathy (IRI total)	0.25	3.62	**p < 0.001**
				Age	0.00	−0.00	p = 0.997
				Sex	0.09	1.54	p = 0.125

Significant values in bold.

**Figure 2:**
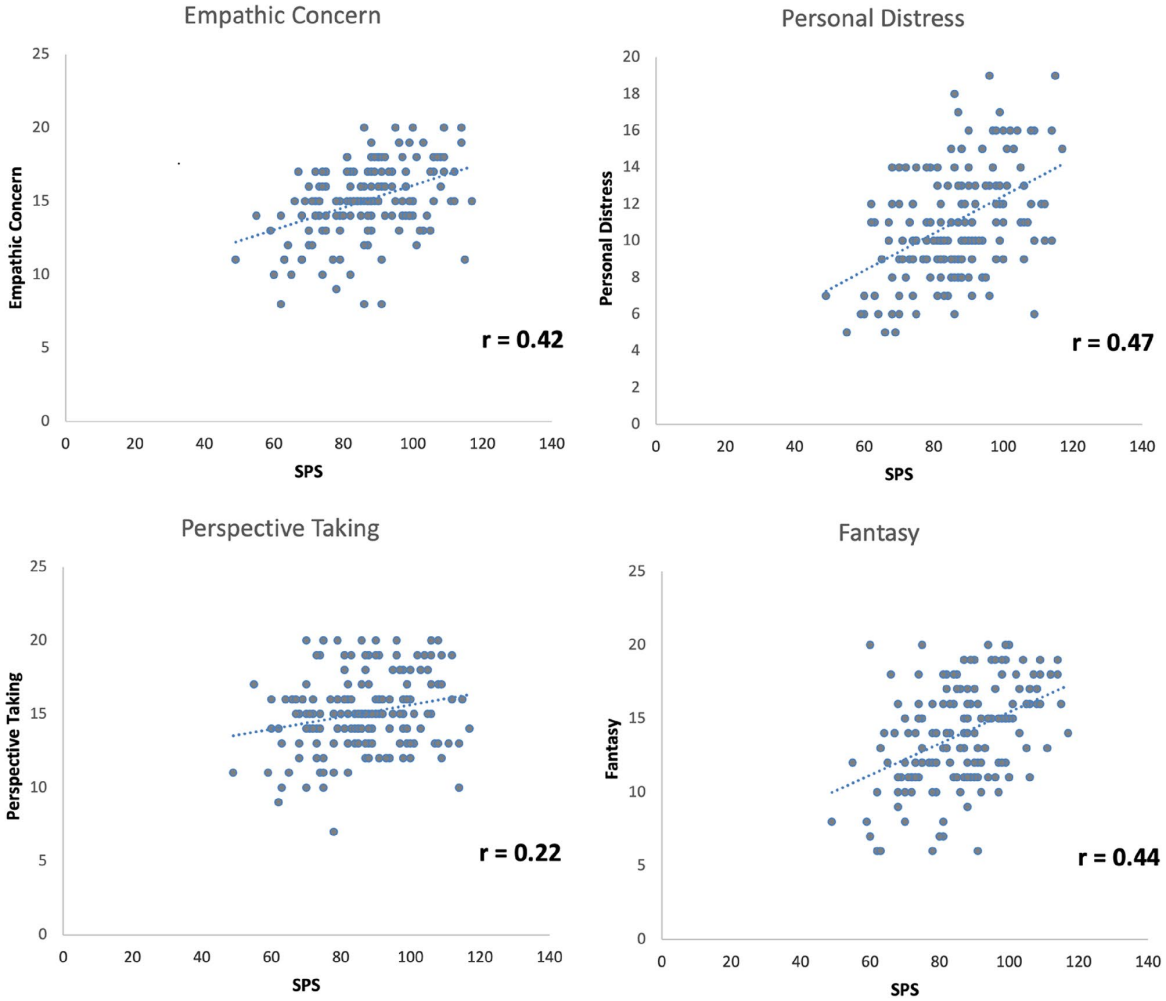
Scatterplots of SPS and empathy measures (IRI). Figure shows significant positive correlations for all empathy subdimensions (Pearson correlations).

To further examine the contribution of empathy more in detail Fig. 2 shows a scatterplot of all four dimensions of the empathy questionnaire IRI with SPS. Pearson correlations show strong positive correlations for all empathy dimensions (empathic concern: r = 0.42, p < 0.001, fantasy: r = 0.44, p < 0.001; personal distress: 0.47, p < 0.001; perspective taking: r = 0.22, p = 0.004). We then computed a second linear regression analysis, in which all four empathy measures went simultaneously into the model. Results revealed a significant model (R = 0.63, adj.R^2^ = 0.38, F(4,164) = 26.59, p < 0.001) and demonstrated that empathic concern, fantasy, and personal distress were strong predictors for SPS (empathic concern: beta = 0.22, p = 0.002, fantasy: beta = 0.25, p = 0.001; personal distress: beta = 0.38, p < 0.001). Only perspective taking as a predictor showed no significant contribution to SPS (beta = 0.08, p = 0.22) (see Table 3 and Fig. 2). Thus, all empathic dimensions but perspective taking were strongly linked to SPS.

**Table 3:** Regression analyses of SPS with empathy personality measures (IRI subdimensions) as predictors.

Model					Coefficients (standardized)		
R	R^2^	Adj. R^2^	ANOVA		Betas	T	Sign
0.63	0.40	0.38	F (4,164) = 26.59, p < 0.001	Empathic concern	0.22	3.11	**p = 0.002**
				Fantasy	0.25	3.50	**p = 0.001**
				Personal distress	0.38	6.07	**p < 0.001**
				Perspective taking	0.08	1.22	p = 0.224

Significant values in bold.

### FMRI results

**Figure 3:**
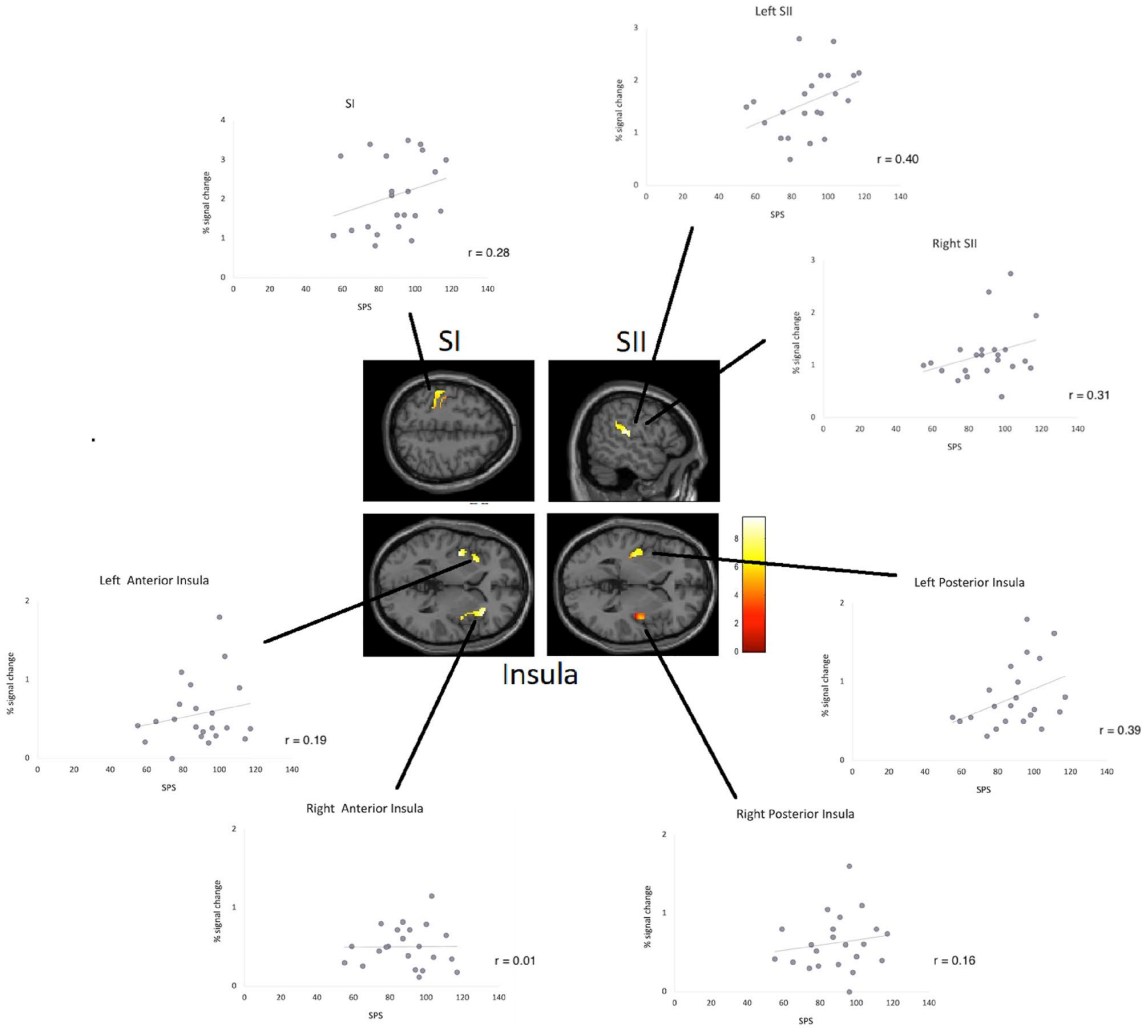
Brain responses when participants were touched by a hand. Scatterplots show relationships between SPS and peak activation of regions of interest (SI, bilateral SII, and insula cortices) (at p < 0.001, uncorrected, for picture display purpose only). Results revealed positive correlations of SPS with SI, SII and left posterior insula, but these correlations hold only for posterior insula when controlling for related personality traits (MNI coordinates, SI: − 50, − 30, 52; right SII: 62, − 16, 20; left SII: − 54, − 28, 20; right anterior insula: 36, 24, 9; left anterior insula: − 30 16 2; right posterior insula: 42, − 8, 2; left posterior insula: − 36, − 18, 12).

We then tested our hypothesis that SPS reflects the brain’s early somatosensory processing by employing an fMRI approach. Brain responses to touch by the experimenter’s hand showed activations in left SI, bilateral SII, and bilateral insula cortices, as expected (p < 0.05, FWE corrected, see Fig. 3). Perceived strength of the passive touch was not related to SPS scores (r = 0.23, p > 0.10, two-sided). In addition, pleasantness of touch was not associated with SPS (r = 0.25, p > 0.10).

Figure 3 shows scatterplots of the relationships between SPS and brain regions related to somatosensory brain activation (left SI, SII, insula). Pearson correlations demonstrate a trend for a positive correlation of SPS scores with brain activity for left posterior insula (Pearson, r = 0.39, p = 0.073; two-sided). Right posterior insula showed no correlation with SPS. Furthermore, left SII showed a trend for a positive relationship with SPS (r = 0.40, p = 0.064). Other brain regions such as SI, right SII, right posterior and bilateral anterior insula cortices (as well as DLPFC, ACC, and medial PFC) failed to reveal significant correlations (or trends, all p > 0.10).

To examine whether the trend for a significant correlation between posterior insula and SPS can still be observed when controlling for personality dimensions associated with SPS (as shown above), we calculated partial correlations using neuroticism and openness, and empathy (sum score) as control variables. Results confirmed the relationship of SPS with somatosensory brain activity in left posterior insula (r = 0.48, p = 0.034). When controlling for those personality dimensions the relationship with left SII correlation coefficients failed to show a significant result (r = 0.39, p = 0.090). Other brain regions computed with partial correlations remain non-significant (all p > 0.10).

Given that empathy (and openness) can be considered as important aspects of the trait SPS, we also computed partial correlation analyses of SPS controlled for neuroticism only. Results showed similar results for relationship of SPS with somatosensory brain activity in left posterior insula (r = 0.51, p = 0.018) and left SII (r = 0.41, p = 0.065) (all other regions p > 0.10).

## Discussion

The present study aimed to test the hypothesis whether higher SPS is reflected by altered brain responses in early somatosensory cortices when simply feeling touch on the palm of the hand. We showed that SPS is closely linked to neuroticism and openness as well as empathy. When controlling for those variables (or for neuroticism only), SPS showed no associations with SI or SII, but was positively related to brain activation in left posterior insula cortex.

Our results confirm previous findings by demonstrating that SPS is positively linked to neuroticism and openness. This is in line with numerous other studies (e.g.,^10,11^. Furthermore, we confirm earlier studies by showing an association of SPS with introversion. For example, Aron and Aron reported similar correlations of SPS with introversion (Pearson’s r = 0.29) and neuroticism (r = 0.54)^2^. However, when calculating a regression analysis, we found that only neuroticism and openness were significant predictors of SPS, not introversion. This seems to be in line with qualitative research showing that not all individuals with high SPS are introverted^2^.

However, the lack of significant results for introversion in this regression analysis might be predominantly caused by the significant predictor empathy. SPS is supposed to be closely associated with empathy personality traits^5^. We found that when adding empathy to the model, introversion did not predict SPS anymore, whereas empathy explains a significant part of the variance in SPS. Hence, individuals with high SPS may not be particularly introverted, but more empathic.

When examining the contribution of empathy in more detail, results revealed that all subdimensions of empathy were positively associated with SPS (especially personal distress), except for perspective taking. Perspective taking describes the capacity to cognitively imagine a situation from the other person's point of view. This finding may be of interest when comparing SPS with autism spectrum disorders (ASD). ASD also describes individuals with high sensory sensitivities, but here impairments in certain parts of empathy, namely the ability to take someone else’s perspective have been reported^30^. This dimension of empathy is reflected by the subscale perspective taking.

The concept of SPS also includes assumptions about the neural underpinnings of this personality trait. It has been argued that SPS describes differences in the way the brain handles sensory information, resulting in variances in somatic sensation^2,5^. Our results do not show any relationships of SPS with somatosensory activations in SI, SII, or anterior insula when processing touch, but suggest that SPS might be associated with brain activation in the left posterior insula (controlled for linked personality dimensions).

The insula can be described as an interface for cognitive and affective processing and has been linked to ownership feelings, sense of agency, and awareness of tactile signals^31^. The insular cortex can be divided into different parts^32^. Most common is the differentiation of an anterior and a posterior part of this brain area. Whereas the anterior insula has been related, for example, to empathy^33^, the posterior part seems to play a role to form perceptual representations of bodily awareness, linked to intensity encoding or localization of somatic and also painful events. For example, a recent study describes pathways from insula to central amygdala to mediate anxiety-related behavior, suggesting that the posterior insular cortex adapts behavior based upon the detection of aversive internal states^34–36^. We speculate that SPS might reflect those processes.

Furthermore, recent research consistently linked insula activity specifically with affective touch. For example, Olausson et al. found that gentle, slow, caressing touch provided by another individual activates C-fibers^20^. These unmyelinated fibers transfer information with slow velocities and project directly to the insula, thereby representing a neural substrate of pleasant and affective touch^37^. Although these C-fibers are predominantly found in the hairy skin (e.g., the forearms)^20^, recent research suggested that also glabrous skin (e.g., the palm of the hand) may include C-fiber mediated touch^38,39^. Furthermore, slow touch to the glabrous skin (the palm of the hand) has been shown to be rated similarly pleasant than touch to the arm (e.g.,^40^). Since a previous imaging study found the insula to be linked to SPS^15^, we argue that our results may indicate that affective touch is processed differently in individuals with high SPS. Thus, general higher processing of affective stimuli in the left insula might explain SPS.

The present work examines SPS with respect to the tactile modality. A previous study had similar aims for the visual domain. Jagiellowicz et al. reported that SPS was linked to stronger brain activations in higher-visual processing when participants were faced with subtle changes in visual scenes^6^. The authors concluded that sensory processing is heightened in individuals with strong SPS scores. In our study we did not find that SPS was linked to enhanced activation in SI or SII. Although our study did not measure tactile perception, it is well-known that activity in SI reflects tactile acuity^41^. Thus, the results of the current study do not support the assumption that SPS is linked to enhanced sensory processing in the tactile domain.

In the present fMRI study we tested correlations of SPS with touch-related brain responses, which were induced simply by touch relative to no touch given by a person the participants did not know. We have to stress that our results represent only a first approach to test whether SPS is linked to processing in somatosensory brain areas. The touch-related brain responses in our study refer to all brain areas that are engaged during processing of touch relative to a baseline control condition (where no touch was applied). Future studies are needed to examine whether other touch events might reveal different relationships of SPS with somatosensory responses. Based on the concept of SPS one could hypothesize that human touch relative to inanimate touch^42,43^ or touch by a friend (relative to a stranger) or touch by someone we like relative to someone we do not like may produce different results (e.g.^28^). Future studies are needed to address these questions.

Further limitations of our study have to be mentioned. First, considering that we present correlational data, the number of participants for the fMRI part of our study is rather small. Therefore, our results need to be replicated by further studies. Second, we do not know whether the role of sex of the touch giver may have affected the correlation between touch and somatosensory activation. Third, we tried to control our results with respect to the Big Five personality dimensions. Future studies are needed to test whether other variables affect SPS or moderate the reported correlation with brain activity.

Taken together, the results demonstrate strong correlations of SPS with neuroticism, openness, and empathy. Furthermore, our study does not show that early somatosensory processing is affected by SPS but suggests that somatosensory processing in posterior insular cortex might be enhanced in individuals with high SPS scores. However, the present study cannot answer the question whether SPS should be treated as a unique personality dimension or as a combination of high neuroticism and empathy or simply as a specific kind of neuroticism. Further research is necessary to assess the potential power of this concept.

## Supplementary Information

Supplementary Information.
