# Supplementary material for: Sensory processing sensitivity and somatosensory brain activation when feeling touch
Source: Sci Rep. 2022 Jul 14;12:12024. doi: 10.1038/s41598-022-15497-9 (PMC9283459; doi:10.1038/s41598-022-15497-9)
Supplement: Supplementary file 1 — Supplementary Information. [file 41598_2022_15497_MOESM1_ESM.docx]

**QUESTIONNAIRE (HSP Scale**)

INSTRUCTIONS: This questionnaire is completely anonymous and confidential. Answer each question according to the way you personally feel, using the following scale:

**1 2 3 4 5 6 7**

**Not at All Moderately Extremely**

| ____ 1. | Are you easily overwhelmed by strong sensory input? |
| --- | --- |
| ____ 2. | Do you seem to be aware of subtleties in your environment? |
| ____ 3. | Do other people's moods affect you? |
| ____ 4. | Do you tend to be more sensitive to pain? |
| ____ 5. | Do you find yourself needing to withdraw during busy days, into bed or into a darkened room or any |

place where you can have some privacy and relief from stimulation?

____ 6. Are you particularly sensitive to the effects of caffeine?

____ 7. Are you easily overwhelmed by things like bright lights, strong smells, coarse fabrics, or sirens close

by?

| ____ 8. | Do you have a rich, complex inner life? |
| --- | --- |
| ____ 9. | Are you made uncomfortable by loud noises? |
| ___ 10. | Are you deeply moved by the arts or music? |
| ___ 11. | Does your nervous system sometimes feel so frazzled that you just have to go off by yourself? |
| ___ 12. | Are you conscientious? |
| ___ 13. | Do you startle easily? |
| ___ 14. | Do you get rattled when you have a lot to do in a short amount of time? |
| ___ 15. | When people are uncomfortable in a physical environment do you tend to know what needs to be |

done to make it more comfortable (like changing the lighting or the seating)?

| ___ 16. | Are you annoyed when people try to get you to do too many things at once? |
| --- | --- |
| ___ 17. | Do you try hard to avoid making mistakes or forgetting things? |
| ___ 18. | Do you make a point to avoid violent movies and TV shows? |
| ___ 19. | Do you become unpleasantly aroused when a lot is going on around you? |
| ___ 20. | Does being very hungry create a strong reaction in you, disrupting your concentration or mood? |
| ___ 21. | Do changes in your life shake you up? |
| ___ 22. | Do you notice and enjoy delicate or fine scents, tastes, sounds, works of art? |
| ___ 23. | Do you find it unpleasant to have a lot going on at once? |
| ___ 24. | Do you make it a high priority to arrange your life to avoid upsetting or overwhelming situations? |
| ___ 25. | Are you bothered by intense stimuli, like loud noises or chaotic scenes? |
| ___ 26. | When you must compete or be observed while performing a task, do you become so nervous or shaky |

that you do much worse than you would otherwise?

___ 27. When you were a child, did parents or teachers seem to see you as sensitive or shy?

HSP Scale © 1997 E. Aron (Aron & Aron, *JPSP*, 1997)
